# Changes in the Bacterial Microbiota in Gut, Blood, and Lungs following Acute LPS Instillation into Mice Lungs

**DOI:** 10.1371/journal.pone.0111228

**Published:** 2014-10-21

**Authors:** Marc A. Sze, Masashi Tsuruta, Shun-Wei Julia Yang, Yeni Oh, S. F. Paul Man, James C. Hogg, Don D. Sin

**Affiliations:** 1 Heart + Lung Innovation Centre at St. Paul's Hospital, University of British Columbia, Vancouver, BC, Canada; 2 Departments of Medicine, Respiratory Division, University of British Columbia, Vancouver, BC, Canada; 3 Pathology and Laboratory Medicine, University of British Columbia, Vancouver, BC, Canada; University Hospital Freiburg, Germany

## Abstract

**Introduction:**

Previous reports have shown that the gastrointestinal (GI) bacterial microbiota can have profound effects on the lungs, which has been described as the “gut-lung axis”. However, whether a “lung-gut” axis exists wherein acute lung inflammation perturbs the gut and blood microbiota is unknown.

**Methods:**

Adult C57/Bl6 mice were exposed to one dose of LPS or PBS instillation (n = 3 for each group) directly into lungs. Bacterial microbiota of the bronchoalveolar lavage fluid, blood, and cecum were determined using 454 pyrotag sequencing and quantitative polymerase chain reaction (qPCR) at 4 through 168 hours post-instillation. We then investigated the effects of oral neomycin and streptomycin (n = 8) on the microbiota at 4 and 24 hours post LPS instillation versus control treatment (n = 5 at baseline and 4 hours, n = 7 at 24 hours).

**Results:**

At 24 hours post LPS instillation, the total bacterial count was significantly increased in the cecum (P<0.05); whereas the total bacterial count in blood was increased at 4, 48, and 72 hours (P<0.05). Antibiotic treatment reduced the total bacteria in blood but not in the cecum. The increase in total bacteria in the blood correlated with *Phyllobacteriaceae* OTU 40 and was significantly reduced in the blood for both antibiotic groups (P<0.05).

**Conclusion:**

LPS instillation in lungs leads to acute changes in the bacterial microbiota in the blood and cecum, which can be modulated with antibiotics.

## Introduction

The bacterial gastrointestinal (GI) microbiota is an active area of research [1]–[4].

GI bacteria have long been suspected to cause some of the inflammation seen during acute lung injury [5] and sepsis [6]–[8]. Some of this inflammation is suspected to be related to bacterial translocation across the GI tract into blood vessels [9], [10]. Further, the GI microbiota has been shown to be an important mediator of lung inflammation [11]. However, it is well known that the most common source of sepsis are lungs (though the putative organism(s) are rarely identified) [12] and it is now well established that lungs, similar to the GI tract, harbor a rich and diverse microbiota that changes with external exposures such as cigarette smoke or disease states such as asthma or chronic obstructive pulmonary disease (COPD) [13], [14]. Despite this knowledge, little is known about the impact of acute lung injury on the lung microbiota and, more importantly, on the bacterial microbiota of the blood and GI tract and whether these changes can be modulated by antibiotics. Our specific aims were first to measure the total bacterial load and bacterial community in the bronchoalveolar lavage fluid (BAL), blood, and GI tract at different time points after acute lung injury. The second aim was to investigate whether any of these changes could be modified with antibiotic therapy. Our final aim was to identify specific bacteria that might be responsible for these changes.

## Methods

### Study Design

All experiments were approved by the University of British Columbia animal ethics committee. C57/Bl6 mice were used and acute lung injury was modeled by directly instilling 2 µg of LPS per gram of body weight [15] (or PBS as controls) into the lungs of these mice through the trachea using a microsprayer Penn-Century Inc. Wyndmoor, PA. First, we performed a time course experiment. At baseline, 5 mice were sacrificed without any instillation as controls. Mice were then assigned to PBS or LPS (n = 3 for each group) and at 4, 24, 48, 72, 96, and 168 hours post-instillation of PBS or LPS, they were sacrificed by exsanguination through the inferior vena cava. Bronchoalveolar lavage fluid (BAL), cecal content, and blood were obtained at each time point to assess the bacterial microbiota. The blood was drawn out with a sterile needle through the inferior vena cava. BAL was sampled by instilling sterile PBS from a syringe into the lungs and suctioning approximately 500 µL out of the lungs and into 1.5 mL microcentrifuge tube. The cecum was sampled by isolating the cecum from the rest of the intestine and using a sterile swab to obtain two cecal samples, which were immediately put into 1.5 mL microcentrifuge tubes. Aseptic technique was used throughout the sampling process.

Next, we investigated whether or not antibiotics modified the acute effects of LPS on the bacterial microbiota by evaluating the microbiome at 4 and 24 hours post-instillation. These were selected based on the findings from the time point experiment. C57/Bl6 mice were pre-treated for 4 weeks with either neomycin (1 g/L) or streptomycin (300 mg/L) [16], which was added to their drinking water and compared to LPS only mice (n = 5 for baseline and 4 hours and n = 7 for 24 hours). Mice in the neomycin and streptomycin groups were divided into PBS and LPS groups and 8 mice in each group were sacrificed at 4 and 24 hours. Blood, cecal content, and BAL were collected for analysis at the time of sacrifice.

### Sample Processing

Cell counts were performed on BAL immediately after sacrifice using a standard cytospin protocol on 100 µL of BAL from each sample. Cells were manually counted using a microscope (Nikon Eclipse E800, Mississauga, Ontario) and converted to counts/mL. Blood and BAL were frozen at −80°C for DNA extraction. Cecal content was placed into a 1.5 mL microcentrifuge tube and immediately frozen at −80°C following sacrifice. DNA extraction was performed using a commercial kit and according to the manufacturer's protocol (Qiagen DNeasy & Stool Extraction Kit). Nanodrop was performed on each sample to assess the quality and quantity of the DNA. Negative controls were water samples subjected to the qPCR or 454 pyrotag sequencing pipeline. Extraction negative controls were samples subjected to the DNA extraction process and then were interrogated using 454 pyrotag sequencing.

### Bacterial Microbiota Assessment

qPCR was used to generate total 16S bacterial counts [13], which were normalized to the DNA and thus expressed as 16S copies/ng of DNA. The only exception was the BAL counts because there was very little total DNA in these samples. Thus normalization to DNA could not be performed and these data are expressed as 16S copies. Before normalization to the DNA concentration, the average 16S counts from the negative controls were subtracted from all samples. Amplicon generation utilized a nested PCR approach. The first round of PCR utilized primers that gave an 881 bp product (27F and 907R) and spanned the hypervariable regions V1–V3. The second round of PCR generated an approximately 550 bp (27F and 519R) product that spanned the hypervariable regions V1–V3, which was sequenced. 454 pyrotag sequencing of the 16S rRNA gene was performed by Genome Quebec on all samples. Sequence clean up and processing was performed using the *mothur* software program and workflow [17], [18]. Alignment and assignment of OTUs to bacterial taxonomies were assigned using the Silva bacteria database [18], [19]. Shannon diversity was used to assess the alpha diversity of the bacterial microbiota. For the time course microbiome experiment, 648,642 total reads were sequenced and 9,564 OTUs were generated. On average there were 5,028±2,388 (mean ± SD) reads generated per sample. In the antibiotic experiment, the microbiome assessment yielded 850, 100 total reads and 5,723 unique OTUs. More information can be found in Table S1, Table S2, and Table S3. On average for the antibiotic microbiome assessment group there were 5449±2511 (mean ± SD) reads generated per sample. More information can be found in Table S4, Table S5, and Table S6. The square root value of relative abundance was used for analysis. Samples were also separated by body site (e.g. cecum, BAL, etc.) and OTUs that were not represented in morethan 5 samples were excluded. The top 100 OTUs from both arms of the study can be viewed in the online supplement as heatmaps according to the three body sites (BAL, Blood, and Cecum) [Figure S1 in File S1 and S2 in File S1].

### Data Analysis

16S counts were analyzed using t-tests and ANOVA and where applicable Bonferroni correction was applied to account for multiple comparisons. Both constrained and unconstrained principle co-ordinate analysis (PCoA) was performed on the microbiota to visualize differences between groups in both the time course and antibiotic experiments. Pairwise Bray-Curtis dissimilarities after square-root transformation of the relative OTU abundances were used to assess statistical significance. Each body site was analyzed separately except when comparing the bacterial microbiota similarity across sites. The latter was accomplished by using SourceTracker [20]. Regression random forest analysis with Boruta feature selection was used to evaluate for specific operational taxonomic units (OTUs) that were important to the total bacterial load and percent polymorphonuclear (PMN) cells in the BAL. Data were analyzed using the R statistical program as well as Prism v. 5 (GraphPad Software Inc. La Jolla California).

## Results

### Time course – Weight and BAL cell count

**Figure 1:**
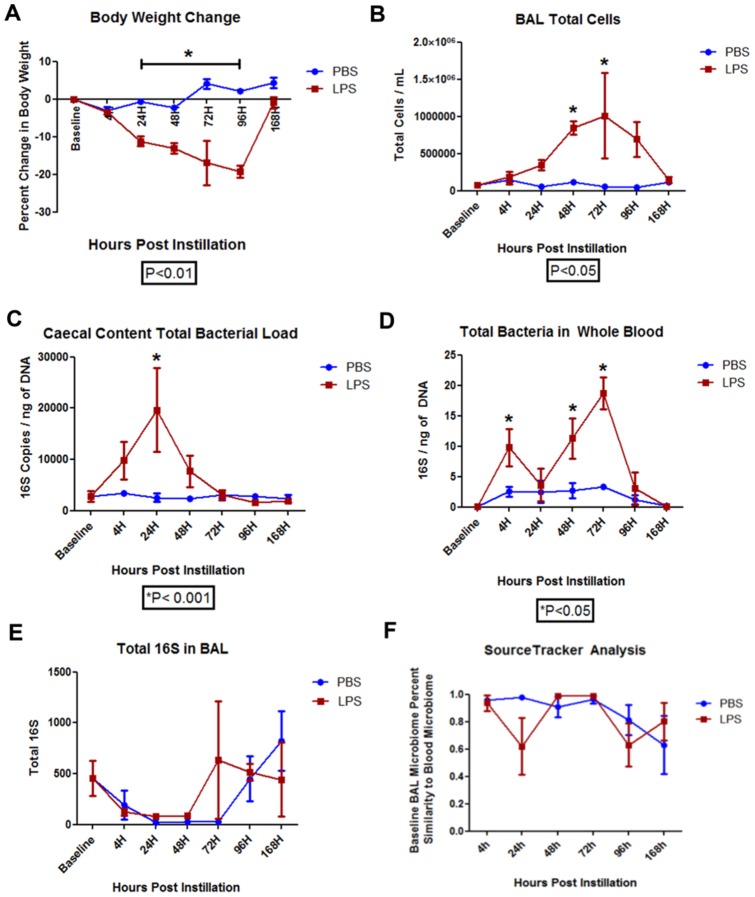
Physiologic and 16S Changes Related to LPS Instillation Directly into Lungs of Mice. **A**) Body weight changed following LPS instillation, P<0.01 from 24 hours through 96 hours versus baseline. **B**) Change in total cells in the bronchoalveolar (BAL) fluid following LPS instillation, P<0.05 from 48 and 72 hours versus baseline. **C**) Cecal bacterial load following LPS instillation, P<0.05 at 24 hours versus baseline. **D**) Blood bacterial load following LPS instillation, P<0.05 at 4, 48, and 72 hours versus baseline. **E**) Total bacterial load in the BAL fluid following LPS instillation. **F**) Sourcetracker analysis of the baseline BAL similarity to the blood bacterial microbiota from 4 to 168 hours post LPS instillation.

There were no premature deaths during the experiments. Following LPS exposure, mice experienced a slight but significant decrease in weight beginning at 4 hours to 96 hours compared with mice which received PBS instillation (P<0.05). The LPS exposed mice demonstrated increased cell count driven largely by neutrophils in the BAL fluid (data not shown) at 48 hours and 72 hours following LPS instillation [Figure 1A & 1B].

### Time course – Total 16S

At 24 hours post-LPS exposure, there was a significant increase in total 16S/ng of DNA in the cecum (P<0.05) [Figure 1C]. Additionally, at 4 hours, 48 hours, and 72 hours there was an increase in total bacterial load in the whole blood in both LPS and PBS groups [Figure 1D]. No significant differences in total bacteria in the BAL fluid were observed throughout the time course study. However, there was a slight trend towards decreased bacterial load at 4 hours, 24 hours, and 48 hours in both the LPS and PBS groups [Figure 1E]. Using Sourcetracker, the peaks in the blood at 4, 48, and 72 hours were determined to be most likely originating from the BAL [Figure 1F].

### Antibiotic Treatment – Weight and BAL cell count

**Figure 2:**
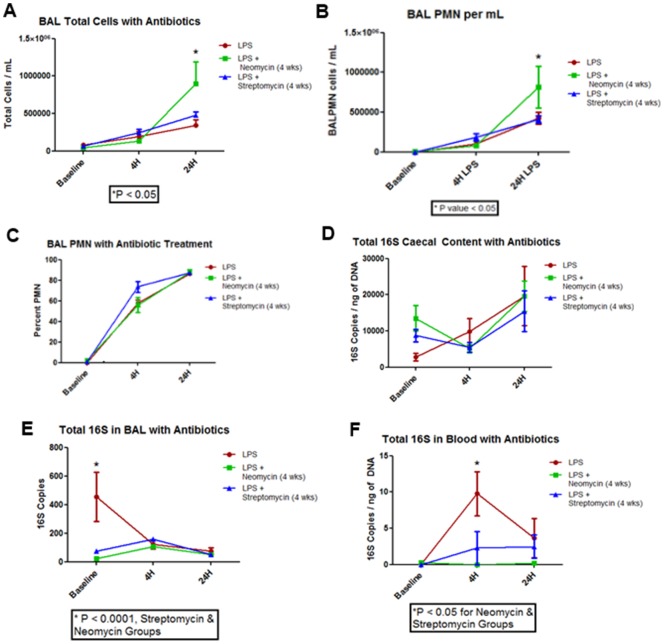
Changes in Lung, Blood, and Cecum Following LPS Instillation in Mice Treated and Not Treated with Antibiotics. **A**) Total cells in the BAL following LPS instillation, P<0.05 at 24 hours for the neomycin group versus LPS alone group. **B**) Total PMN in the BAL fluid following LPS instillation, P<0.05 at 24 hours for the neomycin group versus LPS alone group. **C**) Percentage of PMNs in the BAL fluid following LPS instillation. **D**) Total bacterial load in the cecum following LPS instillation. No significant difference was found between groups (P>0.05) **E**) Total bacterial load in the BAL following LPS instillation, P<0.0001 for the LPS alone group compared with neomycin or streptomycin group. **F**) Total bacterial load in the blood following LPS instillation, P<0.05 at 4 hours for the LPS alone group compared with neomycin or streptomycin group.

All animals completed the 4 hour and 24 hour time course for the antibiotic experiments. No significant differences in weight were found between the antibiotic + LPS groups and the LPS only group (P>0.05). There were a similar number of BAL cells in the streptomycin treatment group and the control group [Figure 2A]. However, at 24 hours post-LPS exposure, there were more cells in the BAL fluid in the neomycin treatment group than the control group (P<0.05), which was driven by an increase in total PMN count (P<0.05) [Figure 2A–C].

### Antibiotic Treatment – Total 16S

Total 16S bacterial load in the cecum did not change with antibiotic treatment (P>0.05). However, treatment with either neomycin or streptomycin increased the total 16S at baseline [Figure 2D]. All groups showed an increase in total 16S bacterial load at 24 hours post-LPS exposure while the antibiotic groups showed a non-significant reduction at 4 hours [Figure 2D]. Both neomycin and streptomycin treatment led to a reduction in 16S count in the blood at 4 hours post LPS instillation compared to LPS exposure alone (P<0.05) with all groups having similar levels by 24 hours [Figure 2F]. This was associated with decreased 16S bacterial load in the BAL fluid for both antibiotic groups compared with the LPS only group (P<0.0001) [Figure 2E].

### Alpha Diversity Changes due to Antibiotics

**Figure 3:**
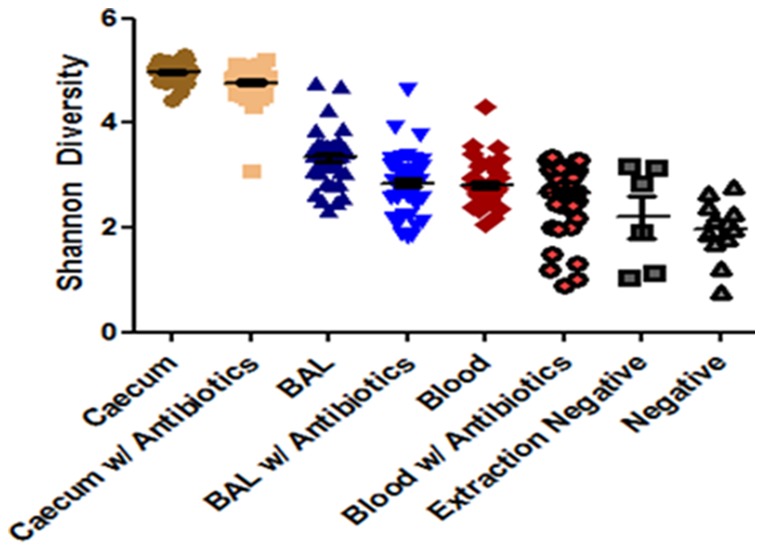
Overall Shannon Diversity by Group. Overall Shannon diversity measures for all samples in the study, P<0.05 for the first three groups versus the extraction negative and negative controls.

**Figure 4:**
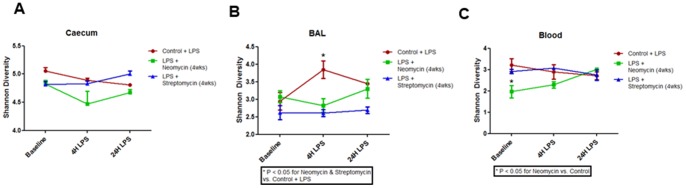
Shannon Diversity by Treatment Group and Location. **A**) Shannon diversity by time and treatment group in the cecum. **B**) Shannon diversity by time and treatment group within the BAL, P<0.05 at 4 hours for the LPS group. **C**) Shannon diversity by time and treatment group within the blood, P<0.05 at baseline for the neomycin group.

Overall Shannon diversity was highest for all samples from the cecum and lowest in the blood samples [Figure 3]. The average Shannon diversity for each compartment was higher than that for both the extraction and negative controls [Figure 3] (P<0.05). The OTU richness was significantly higher only in the cecum in mice treated with antibiotics compared with the extraction and negative controls (P<0.05). There were no differences in Shannon diversity of the cecal samples between 4 hours and 168 hours post LPS instillation [Figure 4A]. Antibiotic treatment caused a slight reduction in the Shannon diversity at baseline but this was not significantly different from the LPS only group (P>0.05) [Figure 4A]. There were also no differences in the Shannon diversity of the cecal microbiota at 4 hours and 24 hours post LPS instillation between the various groups [Figure 4B]. Streptomycin and neomycin treatment significantly lowered the Shannon diversity in the BAL fluid versus the LPS alone group, with this reduction being most evident in the 4 hours post-LPS instillation [Figure 4B] (P<0.05). Neomycin significantly reduced the Shannon diversity in the blood at baseline compared to the other groups [Figure 4C] (P<0.05). However, at 4 and 24 hours, this difference was not present [Figure 4C].

### Beta Diversity Changes due to LPS Instillation

**Figure 5:**
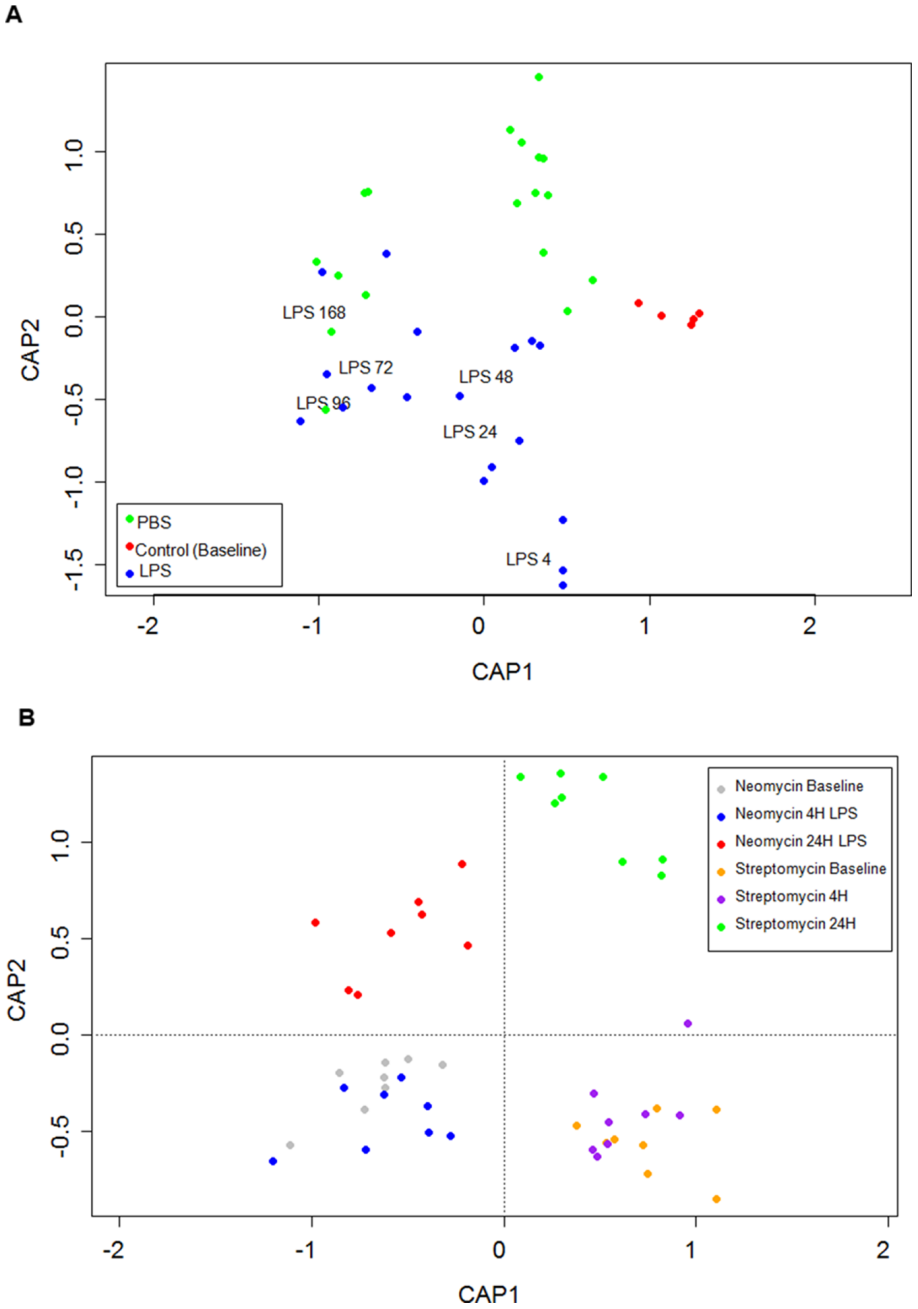
Constrained Principle Coordinate Analysis (PCoA) of the Cecum microbiota. **A**) PCoA analysis of the time course experiment, PERMANOVA, P<0.05. Red are the baseline control mice (n = 5), green are all the PBS timepoints combined (4–168 hours), and blue represents the LPS groups. The labels in the LPS group represent the central point for each timepoint within the LPS group **B**) PCoA analysis of the antibiotic experiment, PERMANOVA, P<0.05. Neomycin baseline is in grey, neomycin at 4 hours is in blue, and neomycin at 24 hours is in red. Streptomycin baseline is in yellow, streptomycin at 4 hours is in purple, and streptomycin at 24 hours is in green. The labels represent the central point for each cluster.

In the absence of antibiotics, PCoA analysis shows that the control, PBS, and LPS groups were significantly different from one another (PERMANOVA, P<0.05) [Figure 5A]. At 4 hours post LPS instillation, the LPS group clustered the furthest away from both the PBS and the control groups [Figure 5A]. However, with passage of time from LPS instillation, the LPS cluster progressively came closer to the control and the PBS clusters [Figure 5A]. Although the alpha diversity did not change in the samples, [Figure S3 in File S1] shows that the BAL microbiota in the LPS only group clustered away from the PBS cluster and from the negative controls and the baseline microbial communities. Figure S2 in File S1 shows that the bacterial microbiota in the blood was different than that of the negative controls samples, though they shared similar Shannon diversity and OTU richness. Further, there appeared to be two distinct microbiomes in the blood samples. One group was composed of samples taken at 4, 48, and 72 hours post-LPS instillation while the other group contained samples taken at 24, 96, and 168 hours post-LPS instillation [Figure S4 in File S1]. The latter group had a microbiome profile similar to that of the baseline samples. These differences in blood microbiota community matched with the peak 16S/ng of DNA load seen in Figure 1D.

### Beta Diversity Changes due to Antibiotics

Figure 4B shows that the antibiotic treatment changed the microbial communities in the cecum. Although at 4 hours post-LPS instillation, the microbial communities were similar to those at baseline, by 24 hours post-LPS instillation, the communities had significantly changed [Figure 4B]. This is in contrast to Figure 4A where the 4 hour post-LPS instillation samples demonstrated the largest changes in bacterial community. Antibiotic treatment reduced the differences between the BAL and blood bacterial communities and the negative control samples [Figure S5 in File S1 & S6 in File S1]. This was consistent with the reduction in total 16S bacterial counts in both these compartments [Figure 2D &2E].

### Identifying Important Bacteria for specific increases in Bacterial Load

**Figure 6:**
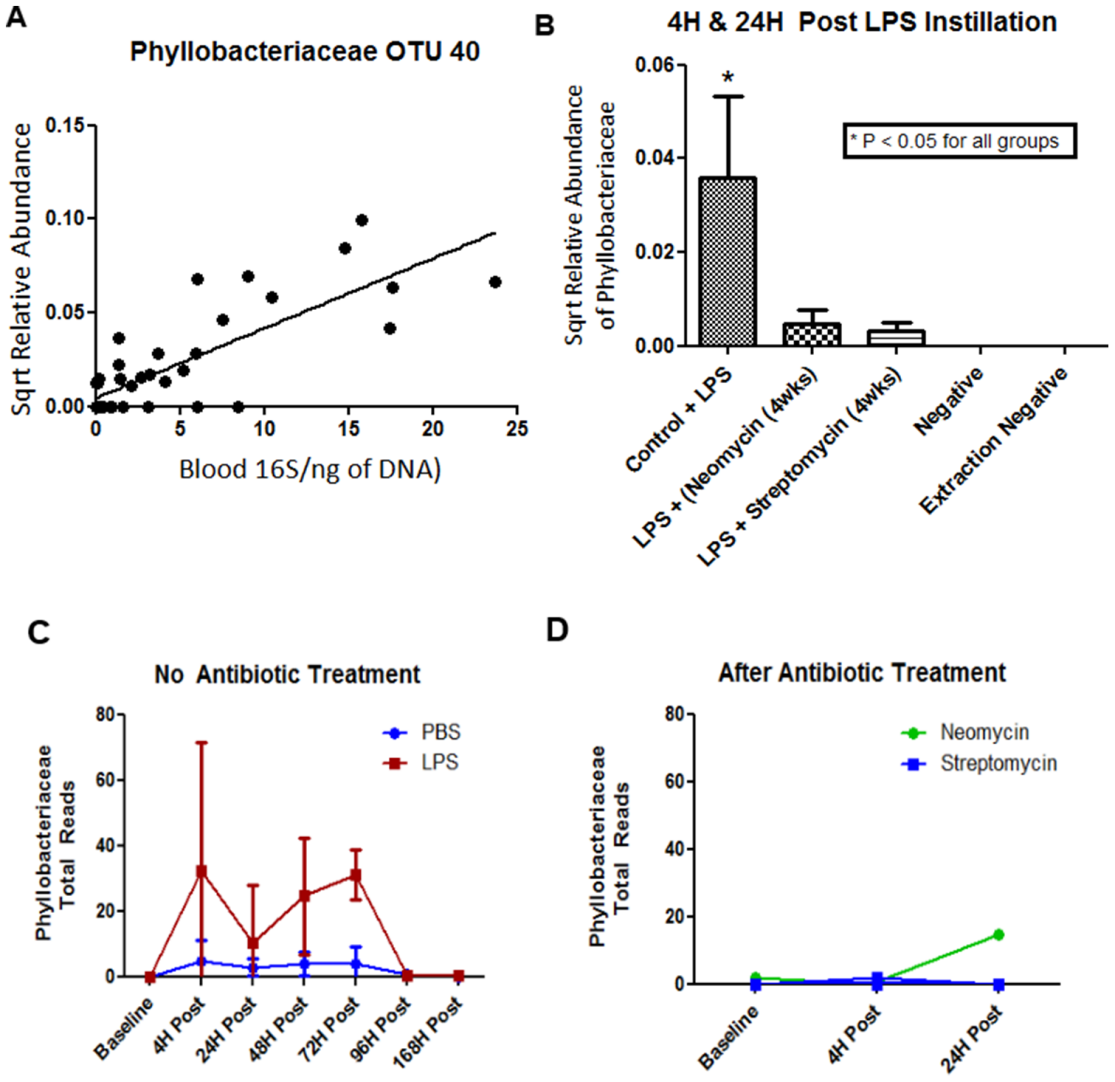
Prominent OTU correlations to Blood Bacterial Load. **A**) Linear regression of bacterial load in the blood and *Phyllobacteriaceae* (OTU 40) relative abundance, Y = 0.0037X – 1.2 (R^2^ = 0.65). **B**) Result on *Phyllobacteriaceae* relative abundance after antibiotic treatment and amount in the control samples, P<0.05 for *Phyllobacteriaceae* against all groups. **C**) Overall total reads detected in the blood for *Phyllobacteriaceae* with either PBS or LPS instillation. Overall P-value for the row factor (LPS versus PBS) was 0.015 and the overall P-value for the column factor (time points) was 0.0025. Post hoc analysis showed no significant difference between the groups. **D**) *Phyllobacteriaceae* after 4 weeks of either Streptomycin or Neomycin pre-treatment.

Using regression random forest with Boruta feature selection we parsed the data for specific OTUs, which were related to increasing total bacterial load in the cecum, blood, and BAL. We also investigated whether or not any OTUs identified in the cecum were related to BAL PMN. 15 OTUs from the cecum were identified that related to total 16S/ng of DNA in the cecum [Table S7 in File S1]. The 14 OTUs in the cecum correlated with PMN BAL counts [Table S7 in File S1]. These OTUs mostly belonged to the *Clostridium* and *Lachnospiraceae* genera. The most significant correlation to total 16S/ng of DNA among the blood OTUs was that belonging to *Phyllobacteriaceae* [Figure 6A & Table S7 in File S1]. When neomycin or streptomycin was given, there was a dramatic reduction in the total number of *Phyllobacteriaceae* which was associated with decreased total 16S/ng of DNA [Figure 6A & 6B]. This particular OTU was not present in either of the extraction negative or water negative controls [Figure 6B] nor was it present in any of the cecal samples. When total reads were analyzed instead of relative abundance there was a significant difference between LPS and PBS groups (P<0.05) but there was insufficient power to determine a difference across time points [Figure 6C]. Upon antibiotic exposure, similar to relative abundance, the total reads dropped and became similar to what was seen in both the negative controls and extraction negative controls [Figure 6D]. Of particular note is that the total reads for *Phyllobacteriaceae* were similar to that seen in total 16S bacterial load in blood throughout the time course [Figure 1D & 6C]. This same bacterium was also found to be one of the important OTUs in BAL total bacteria [Table S7 in File S1] and had a negative correlation with total bacterial load in BAL fluid (data not shown). Further, by performing SourceTracker analysis, we showed that BAL and blood demonstrated the greatest similarity in the microbiota suggesting a common source for both [Figure S7 in File S1].

## Discussion

Acute lung injury with LPS induced the anticipated changes in body weight and influx of neutrophils into the lungs. Unexpectedly, we observed a significant rise in the total bacterial load in the cecum at 24 hours post LPS instillation and a concomitant increase in the total bacterial load in blood at 4, 48, and 72 hours [Figure 1]. We also observed a slight (though not statistically significant) reduction in total bacteria in BAL at 4 to 48 hours post-LPS instillation, with a full recovery at 72 hours [Figure 1]. Importantly, we showed that the microbial communities in BAL and in blood post-LPS instillation were similar suggesting that with acute lung injury, there is translocation of bacteria from the lungs into blood. Oral treatment with neomycin, a selective gram negative antibiotic or streptomycin, a non-specific antibiotic, both of which have poor systemic bioavailability, significantly reduced the baseline bacterial load in BAL and prevented the translocation of bacteria related to acute lung injury. Interestingly, however, neomycin but not streptomycin enhanced the inflammatory response in the BAL fluid related to LPS exposure, suggesting that selective reduction in certain (gram negative) bacteria may increase lung inflammation related to LPS.

These data are in agreement with evolving literature on the human lung microbiota. Contrary to the traditional paradigm, it is likely that the human airways below the vocal cords harbor bacteria and other microbes in a rich microbial milieu. However, environmental irritants such as cigarette smoke or disease states such as asthma or chronic obstructive pulmonary disease, perturb the normal lung microbial flora. We extend these findings by demonstrating that even acute exposure with a single dose of intratracheal LPS disrupts the airway microbiota, leading to translocation of these bacteria into the bloodstream. This process can be abrogated with concomitant antibiotic therapy. We also showed that by 24 hours, the cecal microbiota is also perturbed, leading to a sharp increase in the total bacterial load, which cannot be modified with oral neomycin or streptomycin.

We also showed that while cecal samples harbored the greatest microbial diversity, the BAL samples also demonstrated significant diversity, which could be modulated with antibiotic therapy. In this experiment, we used neomycin and streptomycin because of their poor systemic bioavailability and their different modes of action. Although the Shannon diversity scores were similar between mice treated with neomycin and streptomycin, the PCoA analysis revealed significant differences in beta diversity between the two groups [Figure 5A and 5B] consistent with their different modes of action (one has a broad antimicrobial activity; whereas the other has a selective gram negative bacterial activity). There were other notable differences between neomycin and streptomycin therapy in mice. As noted previously, neomycin enhanced neutrophilia in BAL fluid at 24 hours post LPS instillation. Neomycin but not streptomycin reduced the Shannon diversity in blood at baseline. Together, these data raise the tantalizing prospect that certain (perhaps gram negative) bacteria in the airways may modulate neutrophil influx during acute lung injury or infection. Further work will be needed to validate this hypothesis.

*Phyllobacteriaceae* (OTU 40) was the most prominent bacteria in blood [Figure 6A]. This OTU was highly represented in the BAL fluid at baseline but then decreased following LPS exposure. In blood, however, this OTU increased post-LPS instillation. Treatment with streptomycin or neomycin significantly decreased this OTU in blood [Figure 2 & 6B]. Interestingly, a study recently noted that bacteria in this OTU are cultivatable in blood during severe sepsis related to an acute lung infection [21]. We postulate that this bacteria may translocate during acute lung injury and could cause sepsis in certain settings [21]. However, it should be noted that overall this OTU represented only a small amount of the total abundance observed from sequencing and additional experiments will need to confirm this finding.

There were limitations to this study. First, as our experiments were short-term, we could not evaluate the effects of long-term antibiotic therapy on the lung microbiota. We speculate based on the current experiments that long-term antibiotic therapy would significantly reduce the total bacterial load in lungs and prevent translocation of bacteria into the systemic circulation during acute lung injury. Second, we did not provide antibiotics with selective activity against gram positive organisms. Thus, the impact of gram positive organisms on lung inflammation with LPS injury is uncertain. Third, our mice were healthy and as such we could not determine the impact of antibiotics on the microbiota of disease models such as asthma or COPD.

Notwithstanding these limitations, our study is the first of its kind to demonstrate the impact of acute lung injury on the microbiota of airways, blood, and cecum in healthy mice. Acute lung injury induces a transient translocation of bacteria into blood and causes an acute increase in the bacterial load in cecum. Antibiotic treatment abrogates the translocation but has no effect on the cecum. Selective gram negative antibiotic causes increased influx of neutrophils in the BAL fluid at 24 hours. Although the clinical significance of these observations is uncertain at this point, these data indicate that lung, blood, and cecal microbiotas are very dynamic and are modulated by acute lung injury. Thus, these data suggest that the gut-lung microbial axis is bi-directional.

## Supporting Information

File S1 Figure S1, Heatmap of the top 100 OTUs in all sample sites for the timecourse experiments. Figure S2, Heatmap of the top 100 OTUs in all sample sites for the antibiotic treatment experiments. Figure S3, PCoA of the BAL samples during the time course experiment. Figure S4, PCoA of the Blood samples during the time course experiment. Figure S5, PCoA of the BAL samples from the antibiotic experiment. Figure S6, PCoA of the Blood samples from the antibiotic experiment. Table S7, List of important OTUs identified by regression random forest. Figure S7, SourceTracker analysis of the similarity of the baseline BAL microbiome to all other microbiome samples.
(DOC)

Table S1 List of important parameters for the time course experiments.
(XLS)

Table S2 Time course sequence alignment list.
(XLSX)

Table S3 Time course sequence identification list.
(XLSX)

Table S4 List of important parameters for the antibiotic experiments.
(XLS)

Table S5 Antibiotic sequence alignment list.
(XLSX)

Table S6 Antibiotic sequence identification list.
(XLSX)
