# Supplementary material for: Changes in the Bacterial Microbiota in Gut, Blood, and Lungs following Acute LPS Instillation into Mice Lungs
Source: PLoS One. 2014 Oct 21;9(10):e111228. doi: 10.1371/journal.pone.0111228 (PMC4205020; doi:10.1371/journal.pone.0111228)
Supplement: File S1 — Figure S1, Heatmap of the top 100 OTUs in all sample sites for the timecourse experiments. Figure S2, Heatmap of the top 100 OTUs in all sample sites for the antibiotic treatment experiments. Figure S3, PCoA of the BAL samples during the time course experiment. Figure S4, PCoA of the Blood samples during the time course experiment. Figure S5, PCoA of the BAL samples from the antibiotic experiment. Figure S6, PCoA of the Blood samples from the antibiotic experiment. Table S7, List of important OTUs identified by regression random forest. Figure S7, SourceTracker analysis of the similarity of the baseline BAL microbiome to all other microbiome samples. (DOC) [file pone.0111228.s001.doc]

**Online Supplement:**

**Acute Changes in the Bacterial Microbiome after LPS Instillation into Mice Lungs**

MA Sze, M Tsuruta, J Yang, Y Oh, JC Hogg, DD Sin


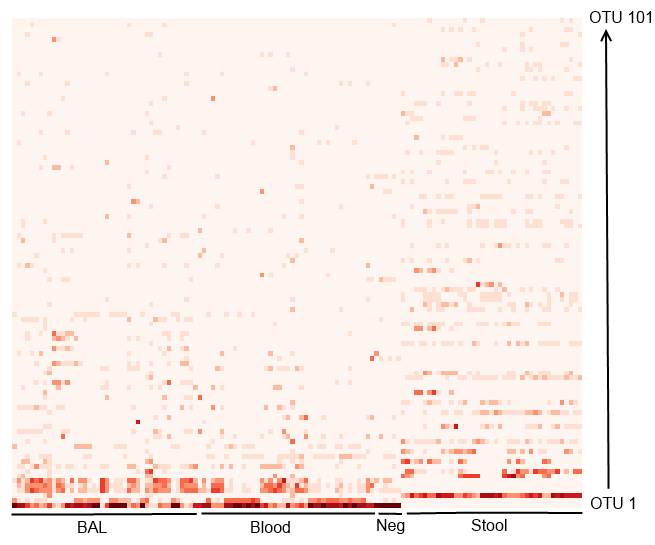


**Figure S1: Heatmap of the top 100 OTUs in all sample sites for the timecourse experiments.**


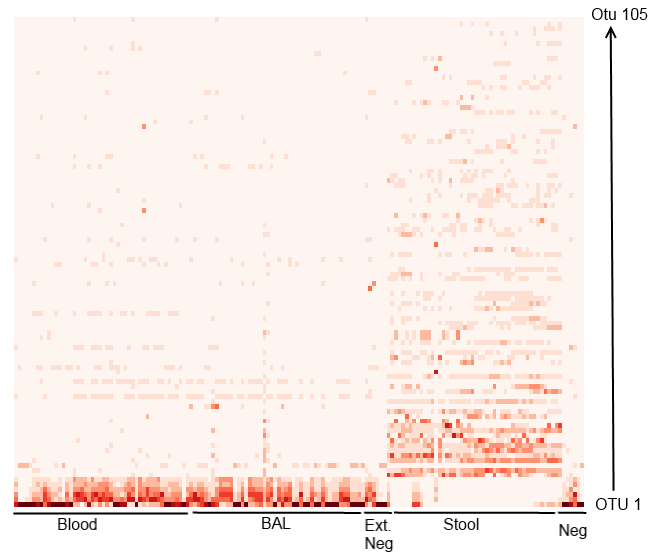


**Figure S2: Heatmap of the top 100 OTUs in all sample sites for the antibiotic treatment experiments.**


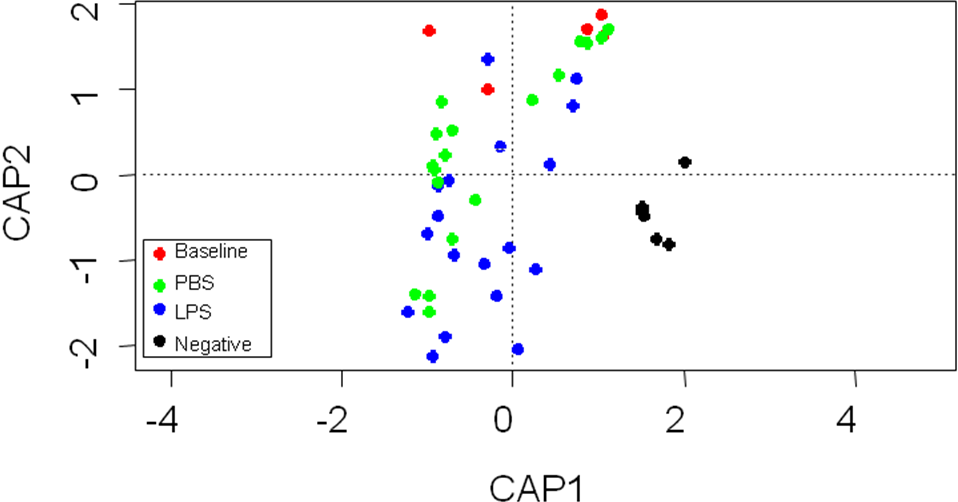


**Figure S3: PCoA of the BAL samples during the time course experiment.**


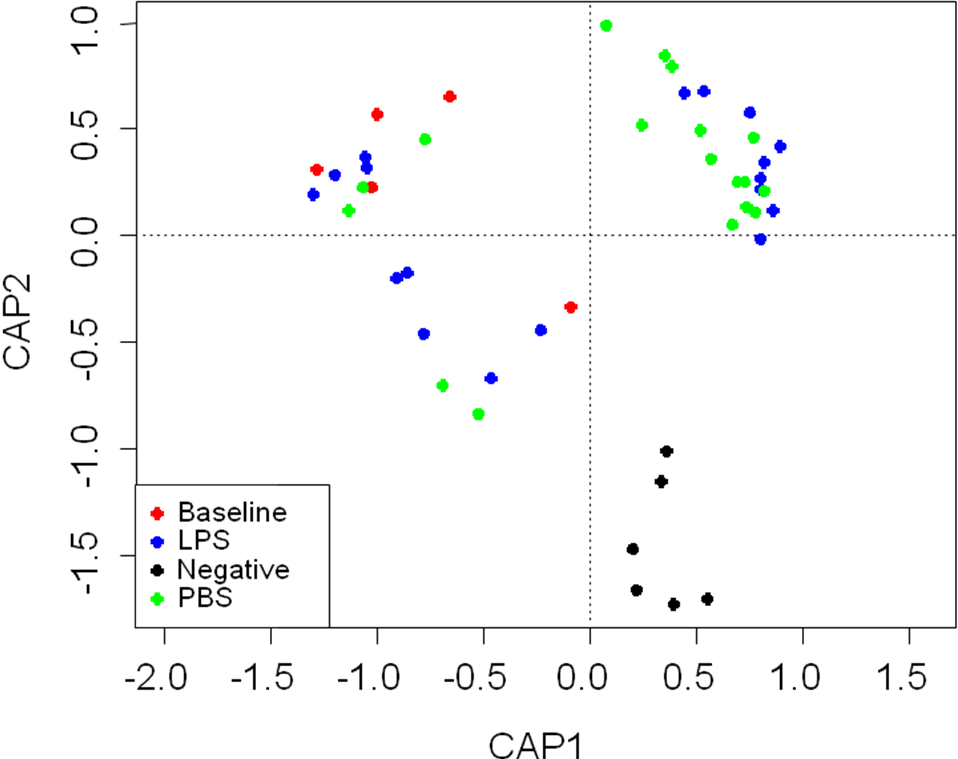


**Figure S4: PCoA of the Blood samples during the time course experiment.**


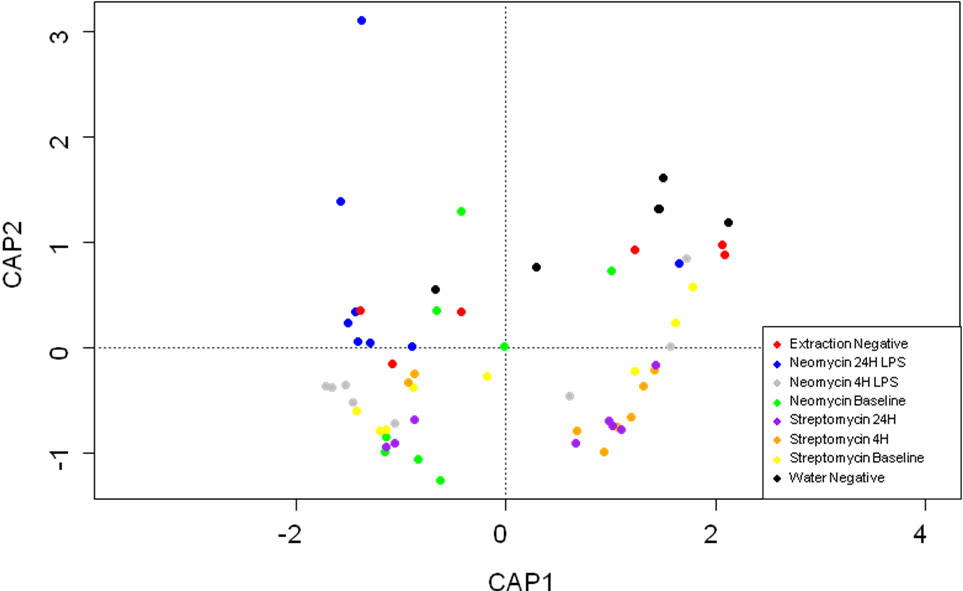


**Figure S5: PCoA of the BAL samples from the antibiotic experiment.**


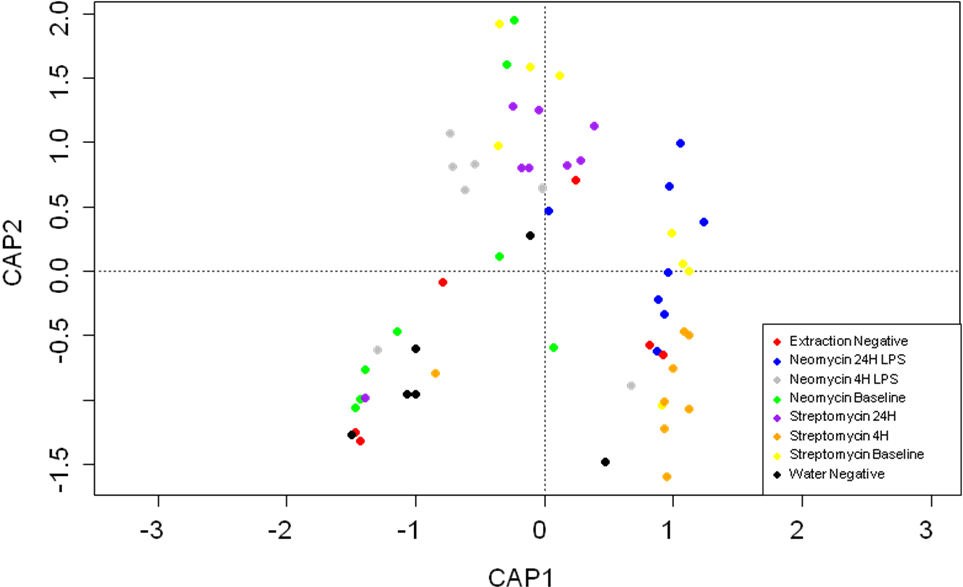


**Figure S6: PCoA of the Blood samples from the antibiotic experiment**

**Table S7: List of important OTUs identified by regression random forest.**

| **Caecal Total Bacteria** | **Importance Factor** | **Blood Total Bacteria** | **Importance Factor** | **BAL Total Bacteria** | **Importance Factor** | **Caecal Bacteria correlating with % PMN BAL** | **Importance Factor** |
| --- | --- | --- | --- | --- | --- | --- | --- |
| Otu0024 | 5.2 | Phyllobacteriaceae | 13.78 | Pelomonas | 4.33 | Otu0076 | 7.52 |
| Otu0037 | 5.47 | Alphaproteobacteria | 5.66 | Bacteroidetes | 4.15 | Clostridium | 3.87 |
| Otu0049 | 5.25 | Phyllobacteriaceae | 3.83 | Jeotgalicoccus | 3.52 | Otu0104 | 3.26 |
| Otu0058 | 3.55 | Acidovorax | 3.46 | Phyllobacteriaceae | 6.54 | Lachnospiraceae | 4.61 |
| Alistipes | 5.36 | Caulobacterleidy | 4.56 | Betaproteobacteria | 10.14 | Clostridium | 6.64 |
| Clostridia | 3.39 | Comamonadaceae | 5.83 | Phyllobacteriaceae | 4.79 | Otu0196 | 4.00 |
| Otu0236 | 3.46 | Bacillales | 3.02 | Pelomonas | 3.62 | Lachnospiraceae | 3.98 |
| Otu0244 | 4.38 |  |  | Burkholderiales | 4.73 | Firmicutes | 3.78 |
| Otu0392 | 5.23 |  |  | Bacillales | 3.23 | Catabacteriaceae | 5.98 |
| Otu0604 | 3.35 |  |  |  |  | Otu0345 | 3.17 |
| Otu0605 | 3.08 |  |  |  |  | Otu0462 | 3.60 |
| Otu0609 | 3.36 |  |  |  |  | Otu0490 | 3.99 |
| Otu0639 | 3.74 |  |  |  |  | Otu1015 | 3.06 |
| Otu0949 | 3.21 |  |  |  |  | Clostridiales | 3.37 |
| Oscillospira | 4.33 |  |  |  |  |  |  |


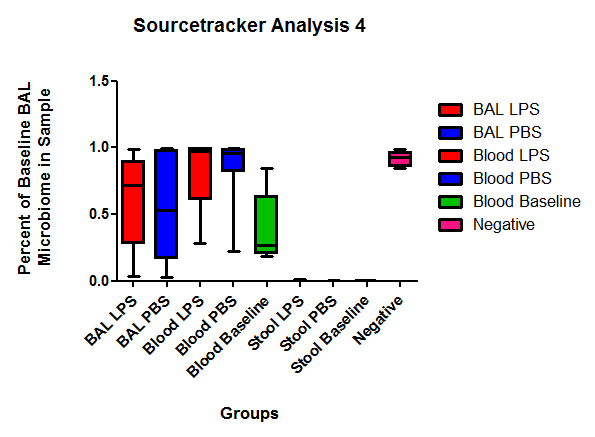


**Figure S7: SourceTracker analysis of the similarity of the baseline BAL microbiome to all other microbiome samples.**
